# Isolation of human β-defensin-4 in lung tissue and its increase in lower respiratory tract infection

**DOI:** 10.1186/1465-9921-6-130

**Published:** 2005-11-04

**Authors:** Shigehisa Yanagi, Jun-ichi Ashitani, Hiroshi Ishimoto, Yukari Date, Hiroshi Mukae, Naoyoshi Chino, Masamitsu Nakazato

**Affiliations:** 1 Third Department of Internal Medicine, Miyazaki University School of Medicine, Miyazaki 889-1692, Japan; 2 Second Department of Internal Medicine, Nagasaki University School of Medicine, Nagasaki 852-8501, Japan; 3 Peptide Institute, Inc. Osaka 562-8686, Japan

## Abstract

**Background:**

Human β-defensin-4 (hBD-4), a new member of the β-defensin family, was discovered by an analysis of the genomic sequence. The objective of this study was to clarify hBD-4 expression in human lung tissue, along with the inducible expression in response to infectious stimuli, localization, and antimicrobial activities of hBD-4 peptides. We also investigated the participation of hBD-4 in chronic lower respiratory tract infections (LRTI) by measuring the concentrations of hBD-4 peptides in human bronchial epithelial lining fluid (ELF).

**Methods:**

The antimicrobial activity of synthetic hBD-4 peptides against *E. coli *and *P. aeruginosa *was measured by radial diffusion and colony count assays. We identified hBD-4 in homogenated human lung tissue by reverse-phase high-performance liquid chromatography coupled with a radioimmunoassay (RIA). Localization of hBD-4 was studied through immunohistochemical analysis (IHC). We investigated the effects of lipopolysaccharide (LPS) on hBD-4 expression and its release from small airway epithelial cells (SAEC). We collected ELF from patients with chronic LRTI using bronchoscopic microsampling to measure hBD-4 concentrations by RIA.

**Results:**

hBD-4 exhibited salt-sensitive antimicrobial activity against *P. aeruginosa*. We detected the presence of hBD-4 peptides in human lung tissue. IHC demonstrated the localization of hBD-4-producing cells in bronchial and bronchiolar epithelium. The levels of hBD-4 peptides released from LPS-treated SAECs were higher than those of untreated control cells. ELF hBD-4 was detectable in 4 of 6 patients with chronic LRTI, while the amounts in controls were all below the detectable level.

**Conclusion:**

This study suggested that hBD-4 plays a significant role in the innate immunity of the lower respiratory tract.

## Background

Bronchial epithelial lining fluid (ELF) contains various antimicrobial substances to protect against pathogenic insult. The antimicrobial components of the ELF are lysozyme, lactoferrin, secretory phospholipase-A2, and antimicrobial peptides, including defensins [1]. Defensins, which are single-chain, strongly cationic antimicrobial peptides with a molecular weight of 3,000–4,500, have broad-spectrum antimicrobial activities against various Gram-positive and Gram-negative bacteria, mycobacteria, fungi, and certain enveloped viruses [1]. Defensins are classified as α-and β-defensins based on the connectivity of their six cystein residues [1]. Human β-defensins (hBDs) are expressed mainly in epithelial cells. hBD-1 is expressed constitutively in the epithelia of the urogenital tract, trachea, and respiratory tract [2-4]. hBD-2 and hBD-3, isolated from psoriatic scale extracts [5,6], are expressed mainly in the respiratory tract, and their expression increases in response to infections and inflammatory mediators [6-11]. In addition, these two hBDs show strong antimicrobial activity against pathogens of respiratory infections, including *P. aeruginosa*, and thus they seem to function in airway mucosal defense [6-11].

hBD-4, a new member of the β-defensin family, was identified by analysis of genomic sequence mapping at chromosome 8p23, where all known α- and β-defensins are clustered [12]. hBD-4 mRNA is expressed in human testis, stomach, neutrophils, lung, and other organs [12], but neither hBD-4 peptide expression in human lung tissue nor its pathophysiological significance in respiratory tract infections has been clarified. We here studied the role of hBD-4 in lower respiratory tract infections (LRTI). We showed the existence, localization, and inducible expression of hBD-4 in response to infectious stimuli. In addition, we determined the concentrations of hBD-4 in human ELF collected by the bronchoscopic microsampling (BMS) method to investigate the significance of hBD-4 in respiratory tract infections.

## Methods

### Peptide synthesis

The reduced peptide of hBD-4, designed by García *et al*. and composed of 37 amino acid residues, was obtained by the chemical ligation method [12]. An oxidative folding reaction of the reduced peptide was carried out in 0.1 M ammonium acetate buffer (pH 7.8) in the presence of reduced and oxidized glutathione (GSH/GSSG) in a molar ratio of 1/100/10 (reduced hBD-4/GSH/GSSG) at 4°C overnight. Reversed-phase high-performance liquid chromatography (RP-HPLC) analysis revealed a single distinct main product, which was purified by preparative RP-HPLC on a YMC C18 column and ion-exchange chromatography on CM-Sepharose. The peptide thus obtained was passed through columns of Muromac and then Sephadex LH-20 to obtain hBD-4 in the acetate form (the yield of the oxidized peptide was 56% based on the reduced peptide). The purity of synthetic hBD-4 was confirmed to be sufficiently high by RP-HPLC, IEX-HPLC, capillary zone electrophoresis, amino acid analysis, sequence analysis, elemental analysis, and matrix-assisted laser desorption/ionization time-of-flight (MALDI-TOF) mass spectrometry (observed m/z was 4367.3, theoretical [M+H]^+ ^= 4367.0). The synthetic products of hBD-2 and hBD-3 were purchased from Peptide Institute Inc. (Osaka, Japan).

### Bactericidal assay

Radial diffusion and colony count assays were used to examine antimicrobial activity [13,14]. We studied the antimicrobial ability of synthetic hBD-4 as well as hBD-2, hBD-3, and penicillin G (Sigma, St. Louis, MO, USA) by radial diffusion assay with *E. coli *strain HB101 and *P. aeruginosa *strain PAO1 (supplied by T. Hayashi, Department of Microbiology, Miyazaki University). Briefly, bacteria were cultured at 37°C overnight in trypticase soy broth (TSB; Nissui Pharmaceutical Co., Ltd., Tokyo, Japan). An aliquot of this culture was transferred to fresh TSB and incubated for 4 h at 37°C to obtain cells in logarithmic-phase growth. Following the precipitation of bacteria by centrifugation at 800 × g for 10 min, the samples were washed with phosphate-buffered saline (PBS) and quantified spectrophotometrically at 620 nm. A culture volume containing 1 × 10^6 ^bacterial colony-forming units (CFU) was then added to 10 ml warm (40°C) autoclaved PBS containing 3.0 g of TSB medium and 1% low electroendosmosis-type agarose. After a rapid dispersion of bacteria, the bacteria-containing agar was poured into a plate to form a uniform layer. Wells measuring 3 mm in diameter were then created in the agar using a gel punch. After 5 μl of each control samples and each diluted peptides to each well, the samples were incubated for 18 h at 37°C. The antimicrobial activity was taken as the difference between the size of the clear zone surrounding the wells containing defensins, penicillin G, and those containing control sample.

The antimicrobial activities of hBD-2, hBD-3, and hBD-4 were also examined by colony count assay using *E. coli *HB101 and *P. aeruginosa *PAO1. Then, 5000 CFU of bacteria was incubated for 2 h at 37°C with defensin in concentrations ranging in tenfold steps from 0.1 to 1000 μg/ml. The final volume of the incubation medium was 50 μl. To measure antibacterial activity more precisely, some series were performed by repeating the analysis with defensin concentrations that ranged in twofold steps from 0.625 to 40 μg/ml. Since the differences in salt sensitivity in the antimicrobial activity of hBDs were previously reported [3,6,7,15], we evaluated the salt sensitivity of the antimicrobial activity of the defensins using two incubation media conditions: 1) a high salt condition (Na^+ ^137 mEq/L, Cl^- ^130 mEq/L, K^+ ^4.2 mEq/L, osmolarity 270 mOsm/kg, pH 7.4) and 2) a low salt condition (Na^+ ^95 mEq/L, Cl^- ^90 mEq/L, K^+ ^25 mEq/L, osmolarity 210 mOsm/kg, pH 7.1). The incubation mixtures were serially diluted, spread on nutrient agar plates, and incubated for 18 h at 37°C. The antimicrobial activity was expressed as the colony reduction ratio, defined as the number of killed bacteria to that of control bacteria.

### Preparation of antiserum

hBD-4 (2.5 mg) was conjugated to bovine thyroglobulin (15 mg) using 1-ethyl-3-(3-dimethylaminopropyl)-carbodiimide HCL (400 mg) as described previously [16], then dialyzed five times against two liters of 0.9% sodium chloride to remove unconjugated material. An antigenic conjugate solution (0.9–3.0 ml) was used to immunize three New Zealand white rabbits by multiple intra- and sub-cutaneous injections. The animals were given booster shots every 2 weeks, then were bled 7 days after each injection. All experimental protocols were approved by the Ethics Review Committee for Animal Experimentation of Miyazaki University.

### Study population

For immunohistochemistry, we obtained human normal lung tissues from 2 patients at surgery: a 38-year-old female with pulmonary mucormycosis and a 70-year-old male with bullae. The patient with mucormycosis also exhibited insulin-dependent diabetes mellitus, while the other patient had no complications that induced an immunosuppressive condition. The patient with mucormycosis was a smoker, and the other patient was not. To evaluate the localization of hBD-4 in chronic LRTI, we also obtained human lung tissue from a 63-year-old female with middle lobe syndrome.

For radioimmunoassay experiments, 6 controls (2 males and 4 females, ranging from 30 to 78 years old, 1 smoker and 5 nonsmokers) and 6 patients (2 males and 4 females, ranging from 64 to 83 years old, all 6 nonsmokers) with chronic LRTI who had persistent productive cough with purulent sputum for more than 6 months were enrolled in this study. The following exclusion criteria were adopted for the patient group: (i) steroids, immunosuppressive drugs, or any antibiotics prescribed within 3 months; (ii) cancer or diabetes mellitus. The pathogens of patients with chronic LRTI consisted of the mucoid phenotype of *P. aeruginosa *in 3 cases and the nonmucoid phenotype of *P. aeruginosa *in 3 cases. The controls underwent bronchoscopy to identify the causes of small solitary peripheral nodules. The final diagnoses of the controls consisted of the healing stage of pulmonary suppuration in 1 case and lung nodule of unidentified etiology in 5 cases. According to the results of the histological study, laboratory data, clinical course, and radiological findings including positron emission tomography, we confirmed strongly that the pulmonary diseases in the 6 controls were all benign. In the controls, no bacterial compounds were detected in samples obtained from the respiratory tract. All controls and patients gave written informed consent to participate in the study, which was approved by the Research Ethics Committee of Miyazaki University.

### Immunohistochemical study

Normal lung tissues from the 2 patients mentioned above, as well as lung tissues with chronic LRTI from a 63-year-old female with middle lobe syndrome, were obtained at surgery for immunohistochemical study. The tissues were fixed in 3.7% formaldehyde in 10 mM PBS (pH 7.2), dehydrated in a graded ethanol series, and embedded in paraffin. Cut sections (3 μm thick) were deparaffinized in xylene, rehydrated in a graded ethanol series, and then washed in Tris-buffered saline containing Tween 20 (TBST; DakoCytomation Co., Ltd., Kyoto, Japan). For antigen retrieval, the sections were incubated in 1 μg/ml proteinase K (DakoCytomation) for 30 min at 37°C and treated with 6% hydrogen peroxidase for 60 min to inactivate endogenous peroxidases. Nonspecific binding was inhibited by an incubation in Protein Block (DakoCytomation) for 3 h at 37°C. Preparations were incubated overnight at 4°C with anti-hBD-4 antiserum at a final concentration of 1/10000. Staining was visualized using the Dako CSA system (DakoCytomation) according to the manufacturer's protocol. Control studies utilized normal rabbit serum or anti-hBD-4 antiserum that had been pre-absorbed with 1 μg hBD-4.

### Radioimmunoassay (RIA) procedure

hBD-4 was radioiodinated by the lactoperoxidase method [17]. The ^125^I-labeled peptide was purified by RP-HPLC using a TSK ODS 120A column (Tosoh Co., Ltd., Tokyo, Japan). RIA reaction mixtures were incubated in 50 mM sodium phosphate (pH 7.4) containing 0.25% N-ethylmaleimide-treated BSA, 80 mM NaCl, 25 mM EDTA·2Na, 0.05% NaN3, 0.1% Triton X-100, and 3.1% Dextran T-40. Diluted samples or standard peptide solutions (100 μl) were incubated for 24 h in 100 μl of antiserum no. 1–4 (final concentration: 1/2,100,000). A solution of the tracer, 16,000–18,000 cpm of ^125^I-labeled peptide in 100 μl reaction buffer, was then added. After 24 h incubation, normal rabbit serum and anti-rabbit IgG goat serum were added for an additional 12 h incubation. Bound and free ligands were separated by centrifugation. All procedures were performed at 4°C. Samples were assayed in duplicate. In the RIA for hBD-4, antiserum no. 1–4 recognized hBD-4 with high affinity at final dilutions of 1/2,100,000 (35% binding). Half-maximum inhibition occurred at 7 pg/tube. The peptide remained detectable at the low level of 0.7 pg/tube. At 50% binding, the respective intra- and inter-assay coefficients of variation were 3.9% and 4.2%. This antiserum did not exhibit any cross-reactivity for human neutrophil peptide-1, hBD-1, hBD-2, or hBD-3.

### Chromatographic characterization of immunoreactive hBD-4 in lung

Normal human lung tissue, isolated as described above for immunohistochemical studies, was heated at 95–100°C for 10 min in a 10-fold volume of water to inactivate intrinsic proteinases. After cooling to 4°C, CH_3_COOH and HCL were added at final concentrations of 1 M and 20 mM, respectively. Following homogenization in a Polytron for 15 min, the homogenate was centrifuged at 18,500 × g for 30 min at 4°C. The resulting supernatant was applied to a Sep-Pak C-18 cartridge (Waters, Milford, MA, USA) pre-equilibrated in 0.5 M CH_3_COOH. Peptides were eluted in 35% acetonitrile (CH_3_CN) containing 0.1% trifluoroacetic acid (TFA). The eluate was examined by RP-HPLC on a TSK ODS SIL 120A (Tosoh Co. Ltd., Tokyo, Japan) column using a linear gradient of 10–35% CH_3_CN containing 0.1% TFA at a rate of 1.0 ml/min for 40 min. All fractions were assayed for hBD-4 by RIA.

### Cell culture and induction of hBD-4 expression

Small airway epithelial cells (SAECs) were purchased from Clonetics and grown to monolayers in tissue culture flasks at 37°C in a 5% CO_2_-humidified atmosphere. SAECs were maintained in SAGM (Cambrex Bioscience Walkersville, Inc., Walkersville, MD, USA). Hydrocortisone and bovine serum albumin were removed from this medium before treatment with stimulants and during the time of the study. All experiments were performed between the third and fifth passages.

For the analysis of hBD-4 peptide expression and release, SAECs were grown in a 175 cm^2 ^flask (Falcon). When 70–80% confluence was reached, SAECs were incubated for 24 h with culture medium alone (control) or medium containing 100 μg/ml *P. aeruginosa-*derived lipopolysaccharide (LPS). After stimulation, 70 ml of each medium (derived from approximately 5 × 10^7 ^SAECs) was collected and centrifuged (3500 rpm, 30 min), then the supernatants were transferred to a new tube and stored at -20°C until use. The cells were washed twice with cold PBS. Then 10 ml of PBS was added to the flask, and the cells were scraped and collected into a centrifuge tube. After centrifugation (3500 rpm, 30 min), the PBS was aspirated off. The cell pellet was frozen in liquid nitrogen, weighed, and heated at 95–100°C for 10 min in a tenfold volume of water to inactivate intrinsic proteases. After cooling to 4°C, CH_3_COOH and HCL were added to the respective final concentrations of 1 M and 20 mM, after which the cell pellet was homogenized in a Polytron for 10 min. The homogenate was centrifuged at 18,500 × g for 30 min at 4°C. Both supernatants and extracts from the cells were applied to a Sep-Pak C-18 cartridge pre-equilibrated in 0.5 M CH_3_COOH. The peptides were eluted in 35% acetonitrile (CH_3_CN) containing 0.1% trifluoroacetic acid (TFA). The eluate was lyophilized, and the residue was dissolved in 0.1 M sodium phosphate buffer (pH 7.4) containing 0.05% Triton X-100. The peptides were then measured by RIA for hBD-4.

### Bronchoscopic microsampling of ELF

Using the BMS method, we obtained ELF from patients with chronic LRTI and controls to measure the concentrations of hBD-4. The BMS probe (Olympus Co., Tokyo, Japan) and sampling procedure were described previously [18]. In brief, after routine premedication, a flexible BF-XT40 fiberoptic bronchoscope (Olympus) was inserted into the lungs. After flushing with air to minimize contamination of the samples, the BMS probe was inserted through the channel into the right lower lobe bronchus. Then the inner probe was advanced slowly into the distal airway, and ELF was sampled by placing the probe gently at a site on the target bronchial wall for 10 seconds. The inner probe was withdrawn into the outer tube, and both devices were withdrawn simultaneously. The wet inner probe was sectioned 2 cm from its tip. Three sectioned probes at one time point from each subject were placed in a preweighed tube and weighed. A dilute solution was prepared by adding 3 ml of saline to the tube and vortexing it for 1 min. The solution was transferred to a new tube and stored at -20°C until use. The probe was then dried and weighed again to measure the ELF volume. The saline-diluted sample (3 ml) was applied to a Sep-Pak C-18 cartridge pre-equilibrated in 0.5 M CH_3_COOH. Adsorbed peptides were eluted in 35% CH_3_CN containing 0.1% TFA. The eluate was lyophilized and assayed by hBD-4-specific RIA. The concentrations of hBD-4 in ELF (hBD-4_ELF_) were determined as follows:

hBD-4_ELF _= hBD-4_BMS _× (3 + ELF volume) / ELF volume,

where hBD-4_BMS _is the measured concentration of hBD-4 in the saline-diluted sample. We also assayed the serum concentrations of hBD-4 in both groups. A serum sample (1 ml) of each groups was collected just before the ELF was obtained. Both ELF and the serum were applied to a Sep-Pak C-18 cartridge pre-equilibrated in 0.5 M CH_3_COOH. Adsorbed peptides were eluted in 35% CH_3_CN containing 0.1% TFA. The eluate was lyophilized and assayed by hBD-4-specific RIA.

### Statisitical analysis

Data were expressed as means ± standard deviations (SD). Differences between groups were examined using the analysis of variance (ANOVA) and Scheffe's test. A *p *value of < 0.05 was considered statistically significant.

## Results

### Antimicrobial activity of hBD-4

**Figure 1:**
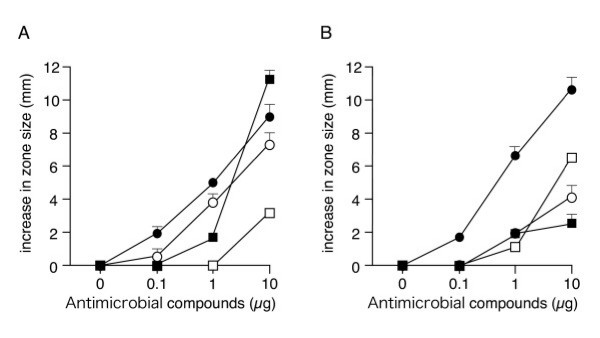
**Antimicrobial activities of hBD-2 (open circles), hBD-3 (closed circles), hBD-4 (open squares), and penicillin G (closed squares). **(A) *E. coli *HB101, (B) *P. aeruginosa *PAO1. An increase in zone size represents the zone size measured at each antimicrobial compound concentration minus the zone size of the central control well (3 mm). Data represent the means ± SD of three independent experiments.

We performed a radial diffusion assay with synthesized defensins and penicillin G. hBD-4 exhibited dose-dependent antimicrobial activity, and this activity was stronger against *P. aeruginosa *than against *E. coli *(Fig. 1). The antimicrobial activity of hBD-4 against *P. aeruginosa *was stronger than that of hBD-2. We next studied the antimicrobial activity of hBD-4 by a colony count assay under two different electrolyte concentrations (Table 1). Under the low salt condition (Na^+ ^95 mEq/L, Cl^- ^90 mEq/L, K^+ ^25 mEq/L, osmolarity 210 mOsm/kg, pH 7.1), the concentration of hBD-4 at which the population of *E. coli *colony was reduced by 50% was 9.1 ± 3.5 μg/ml, which was higher than that for hBD-2 (1.1 ± 0.7 μg/ml). In contrast, hBD-4 had an antimicrobial effect as strong as those of hBD-2 and hBD-3 (1.0 ± 0.5 μg/ml and 0.6 ± 0.2 μg/ml, respectively) against *P. aeruginosa *under the low salt condition (1.3 ± 0.6 μg/ml). The antimicrobial activity of hBD-4, like that of hBD-2, decreased under the high salt condition (Na^+ ^137 mEq/L, Cl^- ^130 mEq/L, K^+ ^4.2 mEq/L, osmolarity 270 mOsm/kg, pH 7.4), although the activity of hBD-3 did not change substantially under these two conditions.

**Table 1:** Concentration of human β defensins effective in reducing 50% colony of bacteria.

	MIC (μg/ml)					
	
	hBD-2		hBD-3		hBD-4	
	
Organism	H-salt	L-salt	H-salt	L-salt	H-salt	L-salt
*E. coli*	26.6 ± 7.6	1.1 ± 0.7	5.7 ± 2.6	4.1 ± 0.8	147 ± 31	9.1 ± 3.5
*P. aeruginosa*	11.6 ± 1.6	1.0 ± 0.5	0.6 ± 0.2	0.6 ± 0.2	>500	1.3 ± 0.6

The bacteria were incubated with defensin in concentrations ranging tenfold in steps from 0.1 to 1000 μg/ml. To measure antibacterial activity more precisely, some values were determined by repeating the analysis with defensin concentrations that ranged in twofold steps from 0.625 to 40 μg/ml. Two incubation media conditions were tested: H-salt was a high salt condition (Na^+ ^137 mEq/L, Cl^- ^130 mEq/L, K^+ ^4.2 mEq/L, osmolarity 270 mOsm/kg, pH 7.4), and L-salt was a low salt condition (Na^+ ^95 mEq/L, Cl^- ^90 mEq/L, K^+ ^25 mEq/L, osmolarity 210 mOsm/Kg, pH 7.1). Values represent the means ± SD of three experiments. (MIC: minimum inhibitory concentration)

### Identification of hBD-4 peptide in the lung

**Figure 2:**
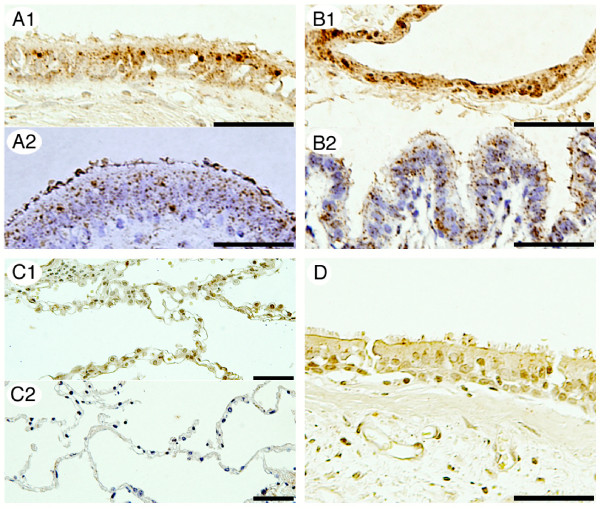
**Immunohistochemical study of hBD-4 expression in the human lung. **For each pair of images, the upper panels (A1, B1, and C1) are the results of the immunohistochemical study of the lung tissue obtained from a 38-year-old female with pulmonary mucormycosis, and the lower panels (A2, B2, and C2) are those obtained from a 70-year-old male with bullae. Immunoreactive cells are present around the bronchial surface (A1, A2) and bronchiolar surface (B1, B2). hBD-4 immunoreactivity is not detected in alveolar epithelial cells (C1 and C2). No immunoreactivity is detected in tissues following preadsorption of antiserum with 1 μg/ml hBD-4 peptide (D). The bar represents a length of 50 μm in all panels.

**Figure 3:**
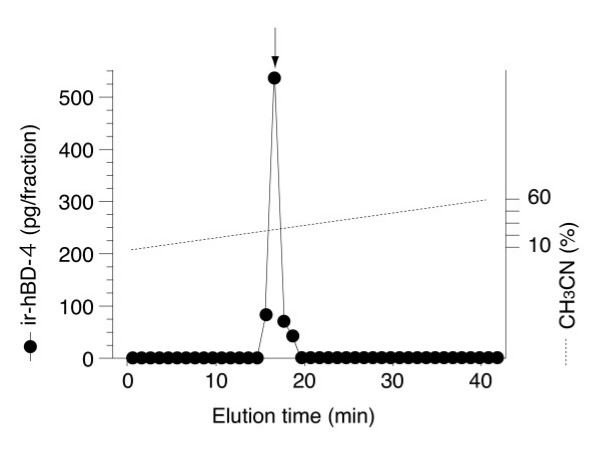
**Representative RP-HPLC profiles of hBD-4 immunoreactivity. **Samples were obtained from 300 mg human lung tissue. Fraction volumes of 0.5 ml were obtained by RP-HPLC using a TSK ODS SIL 120A (4.6 Å × 150 mm) column and a linear gradient of 10–60% CH_3_CN containing 0.1% TFA at a rate of 1.0 ml/min for 40 min. Arrows indicate the elution position of synthetic hBD-4. "ir-hBD-4" on the Y-axis means immunoreactive hBD-4.

**Figure 4:**
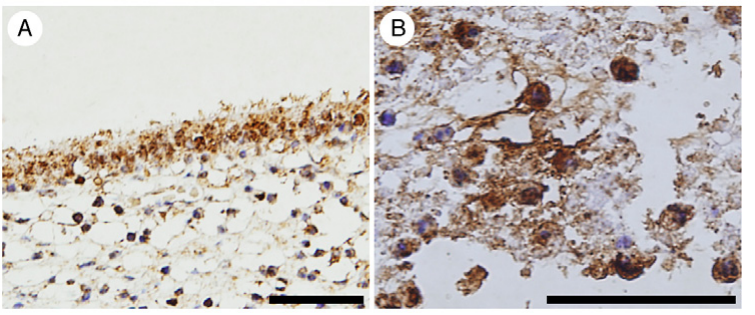
**Immunohistochemical study of hBD-4 expression in patients with chronic lower respiratory tract infection. **hBD-4 immunoreactivity presented in bronchial epithelial cells (A), neutrophils and suppurative exudates within bronchial lumen (B). The bar represent a length of 50 μm in (A, B).

In the two normal lung samples examined, hBD-4-immunoreactive cells were diffusely observed in the bronchial and bronchiolar epithelium (Fig. 2A and 2B, respectively). Airway epithelial cells showed strong and granular cytoplasmic immunostaining. hBD-4 immunoreactivity was not detected in alveolar epithelial cells (Fig. 2C). Tissue immunoreactivity was abrogated by preabsorption of the antiserum with 1 μg/ml hBD-4 peptide (Fig. 2D). Immunoreactive hBD-4 was also identified in the human lung by RP-HPLC combined with RIA (Fig. 3). hBD-4-immunoreactive peaks in the samples were eluted at the same position as the synthetic hBD-4 peptide. We also performed immunohistochemical analysis obtained from one patient with chronic LRTI. Bronchial epithelial cells showed strong and granular cytoplasmic immunostaining (Fig. 4A). Additionally, hBD-4 immunoreactivity was detected in neutrophils and suppurative exudates within the bronchial lumen (Fig. 4B).

### Induction of hBD-4 peptides from lung epithelial cells by LPS in vitro

**Figure 5:**
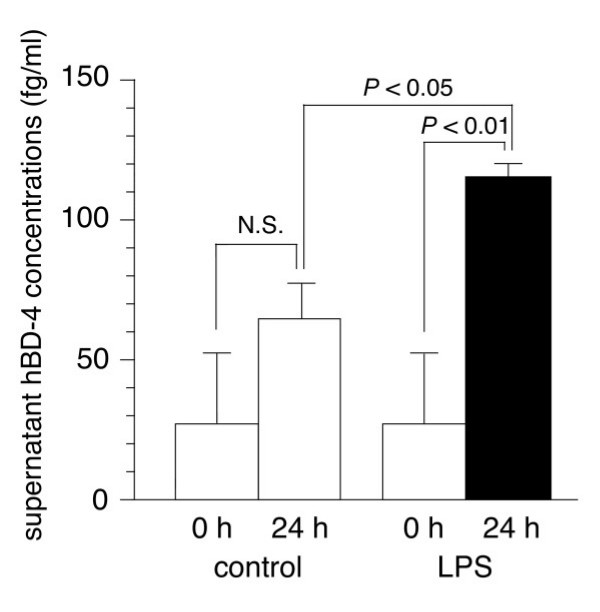
**Expression profiles of hBD-4 SAECs. **hBD-4 peptide concentrations in supernatants of SAECs after 24 h incubation with medium alone (control; open bars), and 100 μg/ml of LPS (solid bar). Values represent the means ± SD of three experiments. (SAECs: small airway epithelial cells, LPS: lipopolysaccharide)

We next assessed whether or not infectious stimuli up-regulate the release of hBD-4 peptide in bronchial epithelial cells *in vitro*. Figure 5 shows the hBD-4 peptide concentrations in the supernatant of SAECs incubated for 24 h with medium alone or with 100 μg/ml of *P. aeruginosa-*derived LPS. The concentrations of hBD-4 peptide released from LPS-treated SAECs were higher than those of untreated control cells (*P <*0.05). Moreover, there was little content of hBD-4 peptide in either the untreated or LPS-treated SAECs (data not shown).

### hBD-4 levels in ELF in patients with chronic LRTI

**Figure 6:**
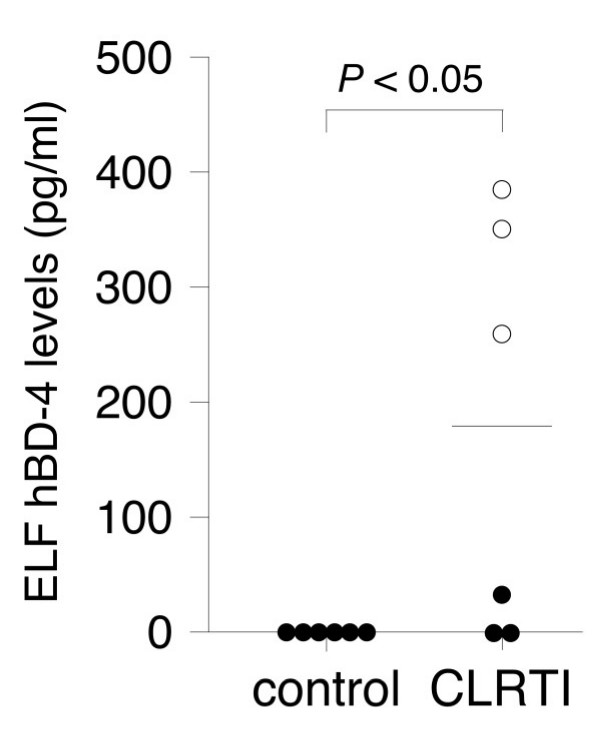
**Epithelial lining fluid levels of hBD-4 in controls (n = 6) and patients with chronic lower respiratory tract infection (n = 6). **In the CLRTI group, open circles indicate patients infected with the mucoid phenotype of *P. aeruginosa*, and closed circles indicate patients infected with the nonmucoid phenotype of *P. aeruginosa*. The horizontal bar represents the mean value. (CLRTI: chronic lower respiratory tract infection, ELF: epithelial lining fluid)

Since β-defensins are expressed constitutively or inducibly in response to infection, we measured the ELF and serum concentrations of hBD-4 in patients with chronic LRTI and controls. ELF hBD-4 was detectable in 4 of 6 patients with chronic LRTI, while the amounts in the controls were all below the detectable level (Fig. 6). The mean ELF concentration of hBD-4 in patients with chronic LRTI was 181.6 pg/ml (range, 0 to 380 pg/ml). All 3 patients infected with the mucoid phenotype of *P. aeruginosa *demonstrated high ELF concentrations of hBD-4, while hBD-4 was not detectable in the ELF of 2 of the 3 patients infected with the nonmucoid phenotype. The serum hBD-4 concentrations of both groups were below the detectable level (data not shown).

## Discussion

The present study indicates that hBD-4 plays a significant role in the innate immunity of the lower respiratory tract. The strong antimicrobial activity of hBD-4 against *P. aeruginosa *rather than against *E. coli*, along with its stronger antimicrobial activity relative to that of hBD-2, underscores the deep involvement of hBD-4 in the innate immunity of the lower respiratory tract. The localization of hBD-4 and other hBD peptides in the bronchial and bronchiolar epithelium also supports that they contribute to the mucosal defenses of the lung [6,7,9,11,15]. We here demonstrated that hBD-4 is induced in the ELF of patients with chronic LRTI, making this study the first investigation of antimicrobial peptide expression in the human respiratory tract *in vivo*. In the present study, the ELF hBD-4 concentrations were not high enough to suppress bacterial proliferation in any patients with respiratory tract infections. However, hBD-4 acts synergistically with lysozyme [12], which is released from neutrophils in *P. aeruginosa *infections [19]. Moreover, hBD-4 is found to have a strong additive effect with hBD-3 [12]. Together, these findings indicate that hBD-4 collaborates with other antimicrobial substances to defend the airway mucosa against *P. aeruginosa *infections.

hBD-4 exhibited salt-sensitive antimicrobial activity. All defensins are strongly cationic, which facilitates their interaction with bacteria and allows the formation of multimeric pores within the negatively charged cell membrane [7]. Previous reports show that the antimicrobial activities of desalted ELF obtained from both cystic fibrosis (CF) and normal xenografts were higher than that of crude ELF obtained from their xenografts. This suggests that high NaCl concentrations inactivate defensin antimicrobial activity by weakening the electrostatic interactions between defensins and the cytoplasmic membrane [20]. The salt sensitivity of hBD-4 strengthens the concept that inactivation of these peptides is one of the major factor in recurrent airway infections in patients with CF.

Compared with the localization of hBD-4 within the cytoplasm of airway epithelial cells in normal lung tissues, hBD-4 immunostaining in lung tissue of chronic LRTI was observed in the bronchial lumen as well as in the cytoplasm of epithelial cells. Also, IHC and ELF findings suggested there was no spontaneous release of hBD-4 into the airway from epithelial cells in the absence of any infectious stimuli. Hence, there is a possibility that hBD-4 in the ELF of the controls was present in amounts too small to be detected. The controls selected here for RIA were not completely healthy. However, pulmonary diseases that are known to induce the expression of defensins, such as malignant diseases, were excluded from the controls. hBD-4 was thought to be released in response to specific stimulation such as infection.

The high hBD-4 levels in supernatant, combined with little content of hBD-4 in LPS-treated SAECs after 24 h, means that SAECs biosynthesized hBD-4 only after being stimulated and released promptly into the extracellular space. It remains unknown whether a direct or indirect action of *P. aeruginosa *is responsible for the biosynthesis and release of hBD-4. Previous reports show that *P. aeruginosa *up-regulates hBD-4 mRNA expression in SAECs [12], but there is a possibility that these phenomena occur indirectly via cytokines produced from airway epithelial cells. However, inflammatory cytokines such as IL-1α, IL-6, interferon-γ, and TNF-α did not induce up-regulation of hBD-4 mRNA expression in SAECs [12]. Therefore, further investigation is needed to clarify the mechanism underlying these phenomena.

Interestingly, the ELF in all patients infected with the mucoid phenotype of *P. aeruginosa *demonstrated high hBD-4 concentrations, while hBD-4 was not detectable in the ELF of 2 of the 3 patients infected with the nonmucoid phenotype. The high hBD-4 levels in ELF may have originated from airway epithelial cells and neutrophils in chronic LRTI, since hBD-4 immunoreactivity was also detected in neutrophils. However, the high hBD-4 levels in ELF could not be explained solely by neutrophilsmediated inflammation because of a significant difference between the mucoid and nonmucoid phenotypes of *P. aeruginosa*. A difference in hBD expression in response to *P. aeruginosa *between the mucoid and nonmucoid phenotypes has also been shown in hBD-2 *in vitro *[10]. The mucoid phenotype of *P. aeruginosa *may contain unique signaling molecules that stimulate respiratory epithelial cells for the production of hBDs. hBD-2 exhibits cytotoxic effects at >50 μg/ml concentrations against airway epithelial cells *in vitro *[21]. And colonization of the mucoid phenotype *of P. aeruginosa *in the respiratory tracts has been related to the progression of bronchial airway pathology [19]. Although it remains uncertain whether or not hBD-4 is cytotoxic to airway epithelial cells, the mucoid phenotype of *P. aeruginosa *can damage the respiratory tracts both directly and via the release of hBDs from bronchial epithelial cells.

The expression of hBD-4 and the release of hBD-4 from bronchial epithelial cells are both up-regulated in response to infectious stimuli [12], while hBD-1 is constitutively expressed in the absence of infectious stimulation [9]. Interestingly, hBD-4 immunoreactivity is not detected in alveolar epithelial cells where hBD-2 is expressed [22]. Furthermore, hBD-4 has specific signal pathways; hBD-4 induction is mediated by protein kinase C, but not by NF-κB or STAT, which are associated with up-regulation of hBD-2 and hBD-3, respectively [11,12,23]. In the present study, hBD-4 as well as hBD-2 exhibited salt-sensitive antimicrobial activity, whereas hBD-3 did not. Finally, although the members of the hBD peptide family have similar amino acid structures, hBD-4 is suggested to play a different role than the other hBDs in the defense against respiratory tract infections.

The hBD-4 peptide exhibited strong antimicrobial activities against *P. aeruginosa*, which is the most virulent pulmonary pathogen because of its intrinsic resistance to multiple classes of antibiotics [24,25]. Antimicrobial peptides have many of the desirable features of a novel antibiotic class. They have a broad spectrum of activity, kill bacteria quickly, are unaffected by classical antibiotic resistance mutations, and have selective toxicity. Although further investigation is required, including *in vivo *study, hBD-4 may be an attractive candidate for a new therapeutic agent against *P. aeruginosa *infection.

## Conclusion

hBD-4 plays a significant role in the innate immunity of the lower respiratory tract. Further molecular analyses of hBD-4 activity will provide a better understanding of the physiological role and pathophysiological significance of this molecule in respiratory infectious disease.

## Competing interests

The author(s) declare that they have no competing interests.

## Authors' contributions

SY evaluated the antimicrobial activity of peptides, performed immunohistochemical study, cultured the SAECs, did the BMS, drafted the manuscript, and participated in the design of the study. HI prepared antiserum, established RIA, and performed RP-HPLC. CN synthesized hBD-4 peptide. JA, YD, HM, and NM conceived the study and helped to draft the manuscript. All authors read and approved the manuscript.
